# Anatomy of posterior cruciate ligament retained in a posterior cruciate ligament retaining total knee replacement: a cadaveric study

**DOI:** 10.1051/sicotj/2018013

**Published:** 2018-09-12

**Authors:** Tarun Goyal, Mukesh Singla, Souvik Paul

**Affiliations:** All India Institute of Medical Sciences, Rishikesh India

**Keywords:** Knee arthroplasty, posterior cruciate ligament, cruciate retaining, ligament, cadaver

## Abstract

*Background*: Recent evidence has highlighted a risk that the majority of posterior cruciate ligament (PCL) is removed while making bone cuts in tibia and femur during total knee replacement surgery. Aim of this cadaveric study is to calculate how much PCL footprint is retained in a PCL retaining prosthesis after routine tibial and femoral cuts are made.

*Methods*: Twelve paired formalin-fixed Indian cadaveric knees were studied. Knees were disarticulated and all soft tissues were circumferentially removed from the tibia and femur. Footprints of antero-lateral and postero-medial bundles were marked on tibia and femur. Proximal tibial and distal femoral cuts were made using standard cutting jigs (Zimmer NexGen LPS). Digital photographs were taken with a magnification marker attached on the bone before and after making the cuts. Area of PCL insertion before and after the bone cuts was measured using software ImageJ (National Institute of Health).

*Results*: Footprint on tibial side was reduced by 9.1%, and on femoral side by 21.8%. Footprint of AL bundle was reduced by 24.3% on the tibial side and by 15.3% on the femoral side. Footprint of PM bundle on tibia was not affected by the bone cut but was reduced by 18.5% on the femoral side.

*Conclusion*: Tibial and femoral insertions of PCL are relatively well preserved after bone cuts are made in a posterior cruciate retaining TKR. There is differential sectioning of antero-lateral and postero-medial bundles of PCL on tibial and femoral sides.

## Introduction

Total knee replacement (TKR) can broadly be of two types, posterior cruciate ligament (PCL) retaining and PCL substituting (or posterior stabilized). PCL is generally intact in osteoarthritic knees and if retained it helps in rollback of femur during knee flexion [1,2]. A PCL retaining prosthesis is based on the principle that the native PCL is retained to provide antero-posterior stability. Cruciate sacrificing prosthesis has additional mechanisms built-in the prosthesis to provide stability and femoral rollback.

About 9–10 mm of bone is removed from tibial surface in proximal tibial cut during a TKR. This removed bone may have some or significant amount of PCL attachment. Recently many magnetic resonance imaging (MRI) based studies have shown that a major portion of PCL is removed during this bony resection [3–7]. This questions our understanding of presently used TKR designs. These radiological findings can be confirmed in a cadaveric study using standard TKR instrumentation. There is lack of cadaveric data on PCL anatomy in cruciate retaining prosthesis and there is only one cadaveric study which concluded that tibial cut removed a large area of PCL insertion from tibia [8]. It is also important to realize that PCL has a broad insertion in anterior portion of medial wall of intercondylar notch of femur. A part of this attachment may also be removed during distal femoral cut. This issue has not been addressed in previous studies. Aim of this cadaveric study is to calculate how much PCL footprint is retained after routine tibial and femoral cuts are made in a PCL retaining prosthesis.

## Material and methods

**Figure 1:**
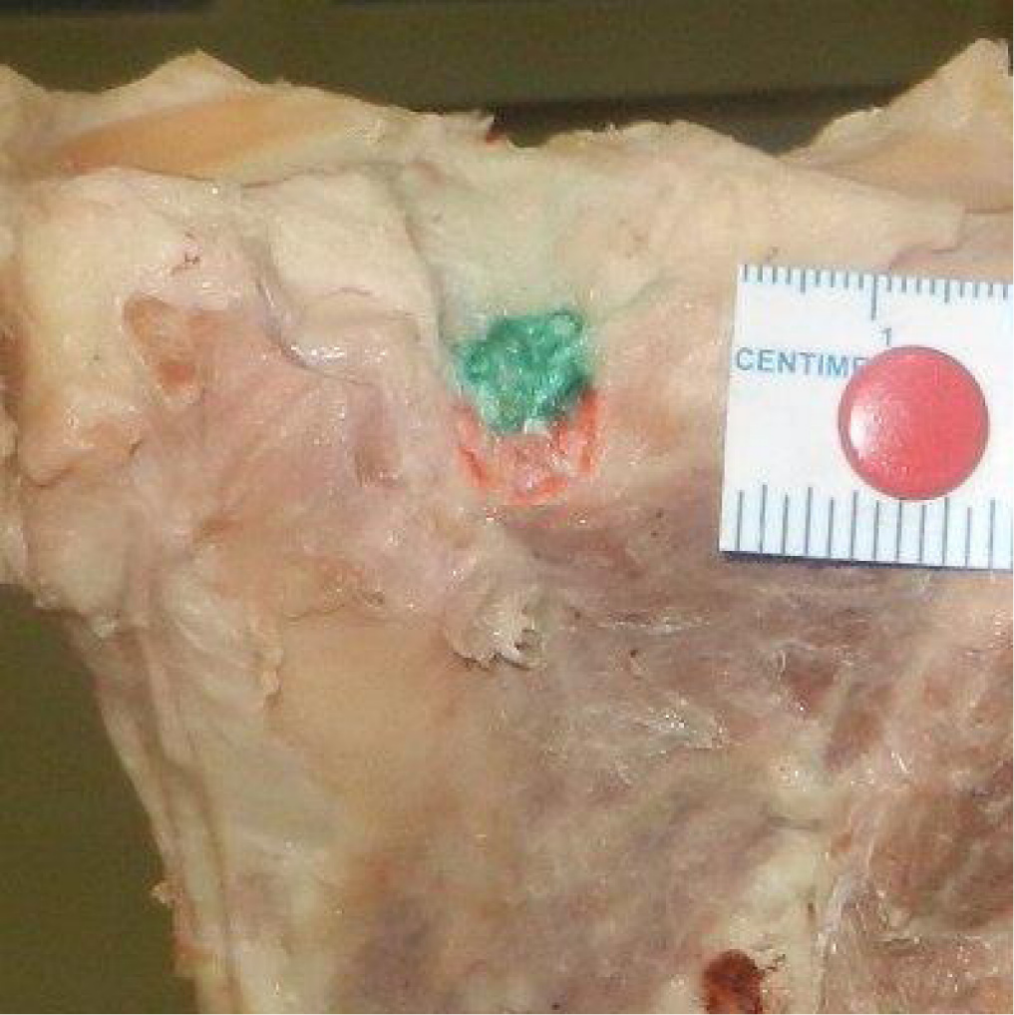
Footprint of PCL on tibia before bone resection. Antero-lateral bundle is marked with green and postero-medial bundle with red.

**Figure 2:**
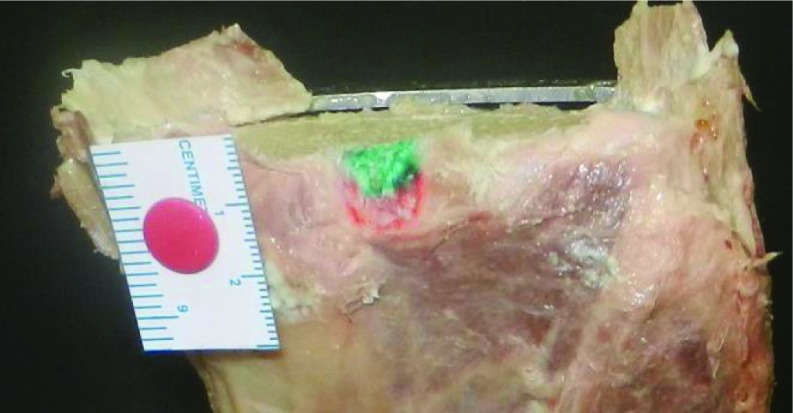
Footprint of PCL on tibia after bone resection. Antero-lateral bundle is marked with green and postero-medial bundle with red.

**Figure 3:**
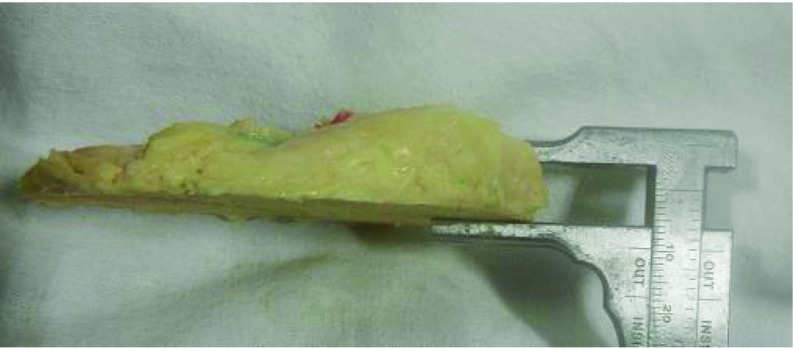
Bone cut from proximal tibia. 10 mm of tone is removed from lateral tibial condyle.

The study was approved by the Institutional Review Board and the Ethics Committee. Twelve paired formalin-fixed Indian cadaveric knees were studied. Mean age of the cadavers was 64.7 years (range = 49–75 years). Seven cadavers were males and 5 were females. We excluded the knees with grade 4 osteoarthritis, according to Outerbridge classification [9]. The tension developing in the PCL was studied as the knee was taken through the range of motion to identify the two bundles- antero-lateral and postero-medial bundles. The knees were disarticulated and all soft tissues were circumferentially removed from the tibia and femur, including the patella and extensor mechanism, carefully preserving the attachment of PCL. The posterior joint capsule, menisco-femoral ligaments, anterior cruciate ligament (ACL), and synovial tissues were carefully dissected to locate the insertion of PCL on tibia and femur. Antero-lateral and postero-medial bundles were identified and footprints of PCL were marked with colored pen. Then proximal tibial cut was made using a standard extra-medullary cutting jig used in TKR (Zimmer NexGen LPS). Ten millimeters of proximal tibia was removed referencing from the lateral tibial plateau, with a 7° of posterior slope. Digital photographs were taken with a marker scale for magnification before and after making the cut (Figures 1–3). Camera was fixed perpendicular to the surface measured. Area of PCL before and after the proximal tibial cut was measured using software ImageJ (National Institute of Health).

**Figure 4:**
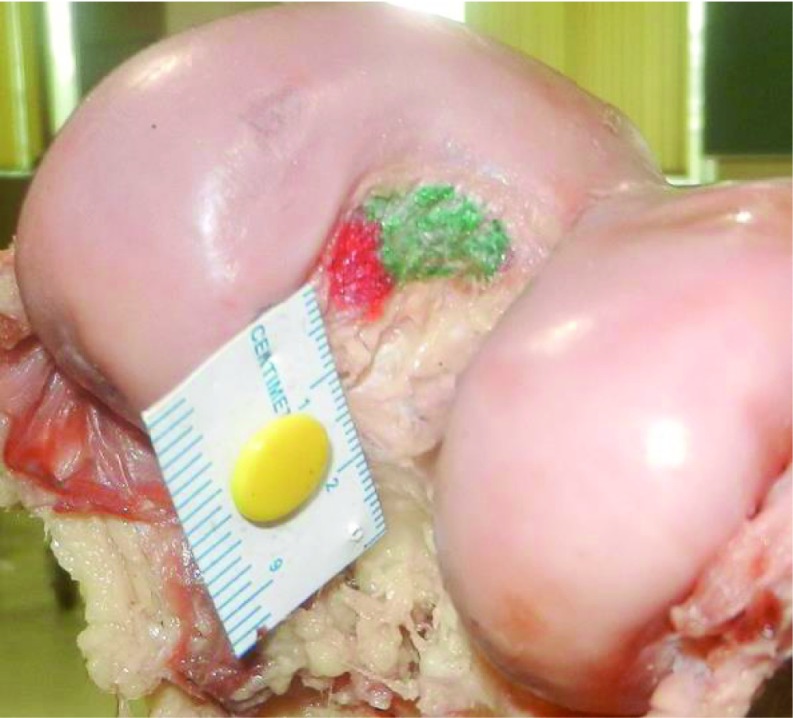
Footprint of PCL on femur before bone resection. Antero-lateral bundle is marked with green and postero-medial bundle with red.

**Figure 5:**
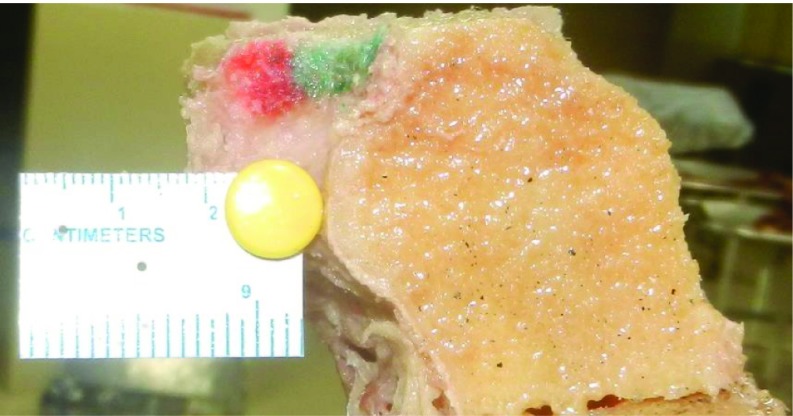
Footprint of PCL on femur after bone resection. Antero-lateral bundle is marked with green and postero-medial bundle with red.

Amount of PCL removed from femur in distal femoral cut was then calculated. PCL footprint was marked on femoral condyle. Distal femoral cut was then made using the standard intra-medullary jig with bone resection of 10 mm and 5 degrees of valgus (Zimmer NexGen LPS). Femur was cut in coronal plane and lateral femoral condyle was removed to obtain a perpendicular view of medial wall of the intercondylar notch for proper measurements. The antero-lateral and postero-medial bundles of PCL were marked. Digital images of the PCL attachment before and after making the bone cuts were taken (Figures 4 and 5).

The size of the tibial and femoral articular surfaces was also calculated using digital photographs with magnification marker. Percentage of area of PCL retained after the cut on tibial and femoral side was correlated with age of the cadavers and the size of tibial and femoral components used using Pearson's correlation. Association between sex of the cadavers and area of PCL retained was studies using students T test. *P*-values <0.05 were taken as statistically significant. All statistical calculations were done using SPSS 19.0 (SPSS Inc., Chicago, IL, USA).

All measurements from the digital photographs were made by two independent observers using the Image J software (National Institute of Health). Mean of the recordings from the two observers was taken as the final value. Inter-observer variability was calculated for total area of PCL insertion on tibia and femur using Intraclass Correlation Coefficients. Intra-observer reliability was calculated on readings obtained on three pairs of cadavers two weeks apart and expressed as Intraclass Correlation Coefficient.

## Results

Mean area of tibial insertion of PCL was 98.1 ± 7.4 mm^2^ (AL bundle 58.0 ± 6.2 mm^2^ and PM bundle 40.1 ± 5.1 mm^2^). Distance of lowermost point of PCL footprint on tibial from the articular surface of lateral femoral condyle was 18.2 ± 3.4 mm. Mean area of femoral insertion of PCL was 172.4 ± 14.3 mm^2^ (AL bundle 98.3 ± 9.5 mm^2^ and PM bundle 70.8 ± 7.9 mm^2^). Distance of deepest point of PCL footprint on femur from the articular surface of medial femoral condyle was 12.7 ± 2.6 mm.

After the cuts were made, mean area of PCL insertion left on tibia was 89.2 ± 6.9 mm^2^ (AL bundle 49.1 ± 6.1 mm^2^ and PM bundle 40.1 ± 5.1 mm^2^). Mean area of PCL insertion left on femur was 134.8 ± 12.3 mm^2^ (AL bundle 74.4 ± 8.3 mm^2^ and PM bundle 60.4 ± 6.9 mm^2^). Footprints on tibial side were reduced by 9.1%, and on femoral side by 21.8% after the bony cuts were made. Footprint of AL bundle was reduced by 24.3% on the tibial side and by 15.3% on the femoral side. Footprint of PM bundle on tibia was not affected by the cut but was reduced by 18.5% on the femoral side. Tibial and femoral footprints of PCL are shown in Figures 1–4.

Mean antero-posterior dimension of tibia measured at the maximum dimension of medial condyle was 39.4 ± 4.4 mm. Mean medio-lateral dimension of tibia at the point of maximum width was 70.8 ± 7.6 mm. There was significant correlation between the area of tibial footprint of PCL and the mean antero-posterior dimension of tibia (*r* = 0.685, *p* = 0.001) and the mean medio-lateral dimension of tibia (*r* = 0.556, *p* = 0.000). Mean length of intercondylar notch as measured along Blumensaat's line was 31.7 mm. Mean total area of medial wall of intercondylar notch was 554.9 ± 37.8 mm^2^. There was significant correlation between the area of femoral footprint of PCL and the length of intercondylar notch (*r* = 0.598, *p* = 0.002) and total area of medial wall of intercondylar notch (*r* = 0.43, *p* = 0.000). The PCL footprint occupied 31 % of the lateral wall of the notch.

There was no correlation between the percentage of PCL insertion preserved and the antero-posterior dimension (*r* = 0.021, *p* = 0.015) and medio-lateral dimensions of the tibial plateau and (*r* = 0.013, *p* = 0.023). Sex of the cadaver was associated with the area of PCL footprint (*p* = 0.020 for tibial side, *p* = 0.035 for femoral side), but was not associated with the percentage area of PCL preserved.

There was good interobserver reliability in measurements of tibial (ICC, 0.912; 95% CI, 0.895–0.968) and femoral PCL (ICC, 0.924; 95% CI, 0.881–0.965) insertion sites. Intraobserver reliability of tibial and femoral measurements was also good (ICC, 0.933; 95% CI, 0.879–0.946 for tibia; ICC, 0.893; 95% CI, 0.846–0.953 for femur).

## Discussion

PCL retaining total knee prosthesis has been successfully used for a long time. Long term results are comparable to PCL substituting designs [10–12]. Advantages of cruciate retaining TKR are lesser bony resection and retention of some biomechanical and proprioceptive role of PCL. PCL is considered to have a crucial role in providing knee stability after TKR and in cruciate substituting designs this function is provided by the post and cam mechanism or a highly conforming and deep polyethylene dish. Choice of the prosthesis is mainly based on preference of the surgeon using it.

There are many controversies surrounding the role of PCL in TKR. Studies have shown that knees in which PCL was sacrificed had equally good outcomes even when a cruciate retaining prosthesis was used [13–15]. But it should be noted that these studies used a highly conforming and dished tibial tray which substitutes for the function of PCL. Thus conclusion on function of a cruciate retaining prosthesis in this situation may not be perfect.

Need of such a cadaveric study was realized because recently many MRI based studies have shown that a major portion of PCL is destabilized after the tibial cut [3–7]. This finding opened up an important debate whether these PCL retaining prosthesis are functioning without an intact PCL. But our cadaveric study supports that majority of the PCL footprint on tibia and femur was intact after the standard bone cuts were made.

One major drawback of radiological studies is that the level of bone resection is relatively arbitrary. These studies use linear measurement of the PCL attachment site on tibial plateau. Linear measurements are not representative of the PCL insertion because the footprint is not symmetrical. We have used two-dimensional areal measurement of the footprint. We used the standard instrumentation which is actually used in TKR as it can better replicate bone cuts. There are no studies on destabilizing effect of femoral cut on PCL. It is important to realize that insertion of PCL on medial wall of intercondylar notch is far anterior and may be damaged while making the distal femoral cut [16].

Our findings are in contradiction to observations made by Feyen et al. [8]. They found that a standard 9 mm tibial cut with 3 degrees posterior slope removed 68.8% of the tibial PCL footprint. We used 10 mm of tibial cut at 7 degrees of flexion and the PCL footprint was reduced only by 9.1%. Thus there is a possible error in marking the PCL footprint in this study. The center of PCL footprint was reported to be much closer to the joint line by the authors. Takahashi et al. [17] found that the centre of antero-lateral bundle was 9.9 mm and centre of postero-medial bundle was 15.1 mm below the posterior border of tibial plateau. This finding was also supported by other cadaveric studies [18,19]. Lee et al. studied tibial footprint of PCL in ten cadavers and found that the tibial footprint is spread between 5.9 ± 1.1 mm and 17.4 ± 4.4 mm below the tibial plateau [18]. Bulk of the PCL is found in the distal part of PCL insertion [19]. Totlis et al. studied tibial footprint of PCL in 30 female patients on MRI preoperatively and intraoperatively measured the length of footprint removed with the tibial cut [20]. They found that 65.1 ± 15.9 % of the footprint length may be lost in the tibial cut. But as already stated linear reduction of the footprint may not correspond to the actual loss of PCL. Onishi et al. performed cruciate-retaining TKR in 20 patients and found that the flexion and extension gaps did not change after the tibial cuts were made [21]. None of these patients had antero-posterior instability at follow-up. They also studied the tibial attachment of PCL on computed tomography and found that more than 50% of the footprint was retained after a 10 mm tibial cut was made using 5 degrees of posterior slope. Another interesting finding in our study is differential sectioning of antero-lateral and postero-medial bundles of PCL on tibial and femoral sides. Whereas PM bundle remains intact on the tibial side even after the cut, 18.5% of it was sectioned from the femoral side. Effect of this finding on functional integrity of PCL needs to be studies in future. This can be studied in more detail using mapping of fibers running between femur and tibia. Hatsushika et al. studies in topographic anatomy of fibers of PCL by dividing it into about 20 bundles [22]. They concluded that topographic anatomy is more sophisticated than simple distribution into two bundles.

Different studies have used different values of posterior tibial slope while making the bone-cut [17–22]. The normal posterior slope of tibia has been found to be 7° by Hofmann et al. [23], 8°–10° by Laskin and Reiger [24], and 14.8° by Chiu et al. [25]. We have used a posterior slope of 7° while making the tibial slope, which is also used in most standard prosthesis.

In conclusion, tibial and femoral insertions of PCL are relatively well preserved after bone cuts are made in a posterior cruciate retaining TKR. The risk of destabilizing the ligament after this surgery is not supported by this study.

## Conflicts of interest

The authors declare that they have no conflicts of interest in relation to this article.
